# Stable 2-year migration on radiostereometric analysis and improved gait speed and function after bicruciate-retaining TKA: a prospective cohort of 25 patients

**DOI:** 10.2340/17453674.2026.46950

**Published:** 2026-10-26

**Authors:** Kelly MILLS, Simon N VAN LAARHOVEN, Koen C DEFOORT, Saskia SUSAN, Petra J C HEESTERBEEK

**Affiliations:** Sint Maartenskliniek, Nijmegen, the Netherlands

## Abstract

**Background and purpose:**

In bicruciate-retaining total knee arthroplasty (BCR TKA) the preservation of both cruciate ligaments necessitates a different tibial tray design with reduced bone–implant interface. These ligaments also influence knee joint force dynamics and may thereby affect component migration. The primary aim of our study was to assess the fixation of BCR TKA up to 2 years postoperatively. Secondary aims included the evaluation of clinical and functional outcomes.

**Methods:**

This prospective study (https://osf.io/zqt8d) included 25 BCR TKA patients. Migration of both components was assessed at different follow-up time points by model-based radiostereometric analysis (RSA) using bone markers. Primary outcome was total translation (TT) and total rotation (TR) at the 2-year follow-up. Secondary outcomes included at-home gait parameters (gait speed, stride length, stride time) assessed with wearable sensors, patient-reported outcome measures (PROMs), and adverse events (AEs).

**Results:**

At the 2-year follow-up, least-squares mean tibial TT was 0.22 mm (95% confidence interval [CI] 0.16–0.28) (tibia) and femoral TT 0.43 mm (CI 0.30–0.57). The least-squares mean tibial TR was 0.53° (CI 0.43–0.63) and femoral TR 0.64° (CI 0.46–0.82). PROMs improved significantly after surgery, with improvements exceeding the predefined minimal clinically important difference (MCID). Gait outcomes revealed a significant gait speed improvement, which remained below MCID, and no significant difference from healthy controls 1 year postoperatively. AEs included 2 intraoperative tibial eminence fractures, 4 cases of persistent postoperative pain, and 2 cases of postoperative oedema with temporary knee stiffness.

**Conclusion:**

RSA analysis of this BCR TKA demonstrated a stabilizing migration pattern that remained below established TKA benchmark values (for different designs), indicating good fixation at 2-year follow-up. The findings were accompanied by an improved PROM, whereas gait speed improvement was below MCID.

Bicruciate-retaining total knee arthroplasty (BCR TKA) aims to more closely replicate native knee kinematics by preserving both the anterior and posterior cruciate ligaments (ACL and PCL). ACL retention has been associated with more natural kinematics and proprioception [1,2]. However, preserving both cruciate ligaments requires a fundamentally different implant design and surgical technique. To accommodate the ACL, the tibial component typically features a horseshoe-shaped design with multiple smaller pegs instead of a central keel. This reduces the bone–cement–implant interface, potentially increasing the risk of implant migration and loosening. In addition, achieving accurate ligament balancing makes the surgical procedure technically more demanding.

Previous radiostereometric analysis (RSA) studies have reported distinct migration patterns for BCR TKA, with stabilization occurring only after 2 years [1,3]. These studies also demonstrated heterogeneous migration patterns with multiple outliers and a relatively high number of device-related adverse events, especially mediolateral instability, possibly attributed to the specific design and the technically challenging procedure of BCR TKAs [1,3]. Furthermore, clinical outcomes, including patient-reported measures and functional performance, have shown variable results [4].

The implant investigated in the present study is a novel BCR TKA design that has not yet been thoroughly evaluated. The tibial component design is different from earlier BCR designs as it features a relatively large and wide keel, with multiple additional smaller pegs. In addition, in this study patient-specific instruments (PSIs) were used to standardize component positioning.

The primary aim was to assess the fixation of both the femoral and tibial components up to 2 years postoperatively using RSA. It was hypothesized that early migration would occur within the first 6 months, followed by stabilization. Given the proposed advantage of more physiological knee function, it is important to also evaluate real-world functional outcomes. Therefore, secondary aims included evaluation of clinical and functional outcomes, including at-home gait assessment and patient- and clinician-reported outcomes.

## Methods

This prospective cohort study was performed in the Sint Maartenskliniek, Nijmegen, the Netherlands, from December 2020 until December 2025 (including 2-year follow-up). The study was conducted and reported in accordance with the Declaration of Helsinki [5], ISO 16087:2013 for RSA, RSA guidelines [6], and STROBE guidelines [7].

### Patients

Patients aged between 40 and 80 years, with non-inflammatory knee osteoarthritis (OA), who were candidates to undergo TKA were eligible to participate in the study. Patients had to be in good general health, with a fully correctable or < 5° fixed varus or valgus deformity of the knee. The main exclusion criteria were BMI > 35, suspicion of ACL or PCL rupture, knee flexion < 90°, fixed flexion deformity > 5° and > 10° extension deficit. A full overview of all inclusion and exclusion criteria can be found in Supplementary Table S1 (see Supplementary data).

Because of the slow rate of inclusions, the following exclusion criteria were removed in an amendment after 13 inclusions and were therefore not applied to the last 12 patients: previous hip replacement surgery, or other joint replacements influencing gait (or when planned in the next 12 months), moderate to severe pain in contralateral knee, hip or ankle (Short Brief Pain Inventory score > 4 on items 3–6 [8]).

Preoperative medial proximal tibial angle, lateral distal femoral angles, and hip–knee–ankle angles were measured on the most recent preoperative long-leg standing radiograph using LAMA software (ImageBiopsy Lab, Wien, Austria). Preoperative radiographic OA severity was assessed on standard radiographs using the Kellgren–Lawrence classification system [9].

### Surgical procedure

All patients received a fully cemented (Palacos R+G, Heraeus Medical, Wehrheim, Germany) Journey II XR implant (Figure 1; Smith & Nephew, Andover, MA, USA), consisting of an oxidized zirconium (Oxinium, Smith & Nephew, USA) femoral component (except 1 cobalt-chromium), titanium tibial baseplate, and 2 polyethylene articular inserts (medial and lateral). In this BCR TKA both the ACL and PCL were retained. Procedures were performed by 2 experienced surgeons (SvL 14 and KD 11 procedures); both had previous experience using PSI and were trained with a cadaver session and > 3 pilot cases or supervised (dual-surgeon) cases before operating independently. Preoperatively, MRI and long standing radiographs were used to design 3D-printed PSI (target: mechanical alignment). Intraoperatively, PSI guides were positioned on the femur and tibia. On the femoral side, PSIs were used to perform the distal femoral cut and position the 5-in-1 cutting block for the remaining femoral cuts. On the tibial side PSIs were used to position the tibial cutting guide correctly. Tibial bone resection was initiated medially with a 3° slope, followed by a trial reduction. Insert thickness and posterior slope were adjusted to optimize PCL tension in flexion, and overall knee balance. Tibial slope was increased by additional resection or decreased by using a definitive insert with 2° less slope. Once balanced, the tibial cut was replicated laterally. The patella was resurfaced for all patients with an onlay oval implant (except 1 inlay implant). Prior to implantation, a maximum of 16 tantalum 1.0 mm RSA beads (Halifax Biomedical Inc, Mabou, Canada) were inserted, distributed between the femoral and tibial bone.

**Figure 1:**
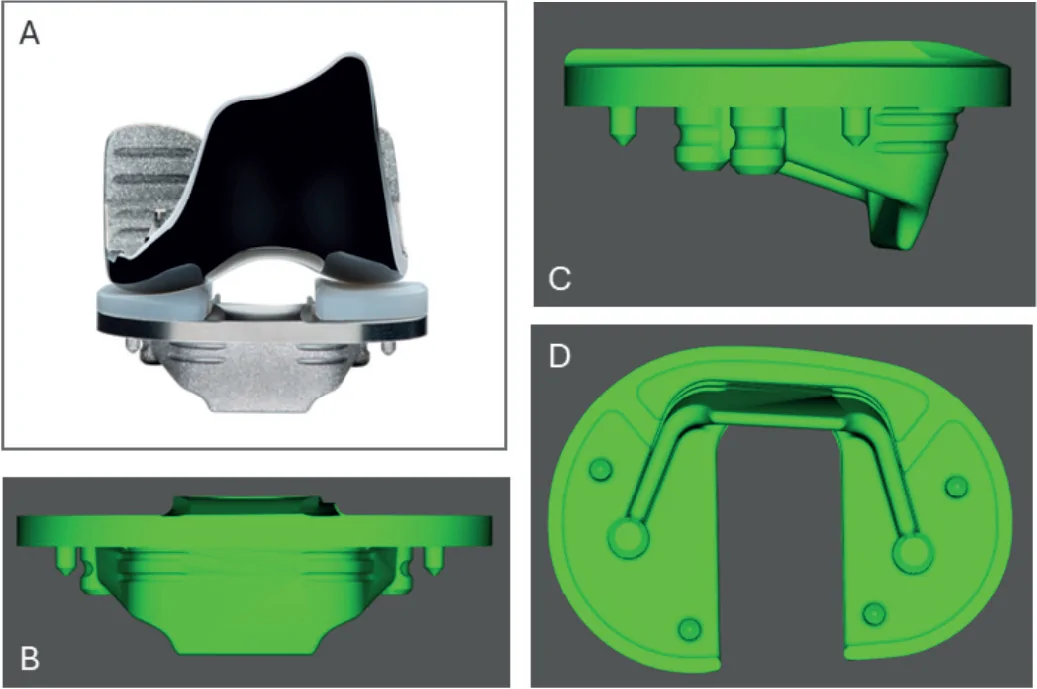
(A) Brochure image of the Journey II XR (Smith & Nephew; USA) BCR implant. (B–D) Different views of the CAD model for the tibial component of the Journey II XR implant; (B) frontal view; (C) lateral view; (D): inferior view.

### Primary outcome

Model-based RSA was used to determine implant migration. RSA images were made in a uniplanar setup (calibrationbox: Medis CarbonBox 004, Medis Specials, Leiden, the Netherlands), with patients in a supine position, using 1 ceiling-mounted and 1 mobile radiograph tube. Digital radiograph detectors were used (Skyplate Large, Philips) with an image resolution of 172 dpi. All RSA measurements were performed using the MBRSA software (MBRSA version 4.2014, RSAcore, Leiden, the Netherlands). Baseline postoperative RSA examinations were performed on the first day after surgery, with 1 exception that was conducted at the end of the same day as surgery, all after weightbearing. The 2-year follow-up measurements were performed 720 (±29) days after surgery. The consistent marker method was used for RSA analysis; a maximum condition number (CN) of 120 and maximum mean error of rigid-body fitting (ME) of 0.35 was applied. The coordinate system was right-handed with the origin located in the geometrical center of the component.

The RSA follow-up times were baseline, 6 weeks (± 1 week), 3 months (± 2 weeks), 6 months (± 2 weeks), 1 year (± 1 month) and 2 years (± 2 months). The primary endpoint was the migration of the tibial component referenced to the (markers in the) bone at 2-year follow-up expressed as total translation (TT = √(Tx^2^ + Ty^2^ + Tz^2^)) and total rotation (TR = √(Rx^2^ + Ry^2^ + Rz^2^)). Secondary endpoints were the femoral TT and TR, as well as migration, of both components, expressed as translation (in mm) and rotation (in degrees) of the components along and around the x, y, and z-axis, and maximum total point motion (MTPM).

At the 6-week follow-up double supine RSA examinations were performed to assess measurement precision, with the RSA setup rebuilt and patients repositioned between examinations. The precision of implant migration measures can be found in Table 1.

**Table 1:** Precision of RSA migration measurements for the tibial and femoral components, assessed through double examinations at 6-week follow-up. Precision was calculated by using the first image as the reference and determining the migration of the second image relative to the first and reported as mean and standard deviation (SD) and median and interquartile range (IQR)

Item	Tibia (n = 25)		Femur (n = 21)	
	Mean (SD)	Median (IQR)	Mean (SD)	Median (IQR)
TT (mm)	0.07 (0.04)	0.06 (0.05 to 0.09)	0.18 (0.12)	0.18 (0.09 to 0.21)
Tx (mm)	0.00 (0.03)	0.00 (–0.02 to 0.03)	0.00 (0.10)	0.01 (–0.03 to 0.07)
Ty (mm)	–0.01 (0.03)	–0.01 (–0.02 to 0.00)	0.01 (0.07)	0.02 (–0.02 to 0.04)
Tz (mm)	0.01 (0.07)	0.01 (–0.05 to 0.05)	0.01 (0.18)	–0.03 (–0.10 to 0.11)
TR (°)	0.20 (0.11)	0.19 (0.11 to 0.21)	0.26 (0.15)	0.23 (0.14 to 0.35)
Rx (°)	0.07 (0.16)	0.04 (–0.00 to 0.17)	0.03 (0.21)	0.04 (–0.05 to 0.14)
Ry (°)	–0.04 (0.13)	-0.07 (–0.11 to 0.02)	0.01 (0.20)	0.05 (–0.06 to 0.15)
Rz (°)	–0.00 (0.07)	0.01 (–0.03 to 0.03)	0.00 (0.10)	–0.01 (–0.08 to 0.05)
MTPM (mm)	0.16 (0.06)	0.14 (0.12 to 0.20)	0.33 (0.19)	0.30 (0.18 to 0.40)

### Secondary outcomes

Mobility at home was measured by measuring daily-life walking, preoperatively and at 1 year and 2 years’ follow-up, using wearable Inertial Measurement Units (IMUs; APDM Wearable Technologies, Portland, OR, USA). 2 of those IMUs were embedded in socks and 1 was placed on the lower back using a belt. For a detailed description of these IMUs see Boekesteijn et al. [10]. Patients were asked to wear the sensors for 5–7 consecutive days, starting in the morning before beginning their daily activities. A control group of 25 healthy individuals, selected out of the same cohort that was included in the study by Boekesteijn et al. [10], were sex and age matched to the patient cohort (mean age difference 1.8 [SD 1.4] years). The raw sensor data was processed using the algorithm described by Shah et al. [11] to extract gait bouts. Gait bouts consisting of ≥ 4 consecutive strides were processed using the Mobility Lab algorithm (APDM, Portland, OR, USA) to calculate gait speed, stride time, and stride length [12]. For each patient a normalized frequency distribution of gait speed was constructed. Outcome parameters for gait performance were real-world gait speed (dominant peak of this distribution), maximum gait speed (95th percentile), stride length, and stride time. Given non-normal distributions, median stride time and stride length were calculated per participant.

Patient-reported outcome measures (PROMs) were scored using electronic surveys sent through Castor Electronic Data Capture (2025; https://castoredc.com) at baseline and 3-month, 1-year, and 2-year follow-up. These surveys contained the following questionnaires: Knee Injury and Osteoarthritis Outcome Score Physical Function (KOOS-PS) [13], Oxford Knee Score (OKS) [14], Numeric Rating Score (NRS) for pain during rest and activity, the Forgotten Joint Score (FJS), and EQ-5D-5L questionnaire [15]. The Knee Society Score (KSS) [16], consisting of a clinical and functional score, was scored by the research nurse. Device-related adverse events (AEs) were noted until the last follow-up.

PROM data was compared with available minimal clinically important differences (MCIDs) from the literature: KSS clinical score of 9 points and functional score improvement of 10 points [17], KOOS-PS improvement of 8 points [18], OKS improvement of 7 points [19], NRS pain decrease of 1.24 points [20], and FJS improvement of 18 points [21]. MCID for gait speed change was 0.10 m/s [22].

### Statistics

A sample size of 25 patients was chosen, as RSA’s high accuracy allows early implant migration to be assessed with as few as 15–25 subjects [23]. This balances study feasibility with the minimum number required for conducting a meaningful RSA study (ISO 16087:2013).

All outcomes (RSA migration, gait outcomes, and PROMs) were analyzed using linear mixed-effects models with time as fixed effect and subject as random intercept to account for repeated measurements within individuals. MTPM values were right-skewed and were therefore log-transformed prior to statistical analysis. Least-squares (LS) means with 95% confidence intervals (CI) were calculated for all (back-transformed for MTPM). To assess differences between BCR and healthy controls a combined group–time variable was created, and BCR values at each time point were compared with healthy controls at baseline. All linear mixed-effects models were fitted using restricted maximum likelihood (REML) to handle missing data under the missing-at-random assumption. Assumptions were checked visually via residual and Q–Q plots.

To allow for comparison with existing RSA literature, medians and interquartile ranges (IQR) were also reported. MTPM was compared with existing RSA TKA benchmarks: MTPM_6-months_ < 0.5 mm [24], MTPM_1-year_ < 0.5 mm [25], ∆MTPM_6-12 months_ < 0.2 mm and ∆MTPM_1-2 years_ < 0.2 mm [24,26]. The estimated maximal MTPM (MTPM-Emax) was calculated using the available R script (https://github.com/elaende/MMRSA) and compared with the available benchmark (MTPM-Emax < 1.3 mm for a safe implant) [27]. The constant k is the time in months at which half the MTPM-Emax is reached but it is not used as a predictor for safe vs unsafe implants.

Body mass index (BMI) was compared between the BCR patients and healthy controls using an independent t-test.

All statistical calculations were performed using R (version 4.5.1; R Foundation for Statistical Computing, Vienna, Austria) in Rstudio (version 2025.09.1; Posit Software, Boston, MA, USA), using the packages lme4, lmerTest, emmeans, tidyverse, performance, and see. A P value < 0.05 was considered statically significant.

### Ethics, registration, funding, data sharing, and disclosures

Ethical approval was obtained from CMO Regio Arnhem-Nijmegen (March 26, 2020, NL71885.091.19) and the study was registered in the Open Science Framework (https://osf.io/zqt8d). All patients were screened and informed by their treating orthopedic surgeon and asked for consent to be contacted by the research nurse. If patients agreed to participate written informed consent was given. A patient representative was involved in the development of the patient information letter. The investigator-initiated study was funded by Smith & Nephew, but they had no involvement in the creation of the protocol, conduct, analysis, or reporting of the results for the study. The datasets gathered and analyzed in this study are not publicly available due to privacy regulations under the Dutch General Data Protection Regulation (AVG), but anonymized output data is available from the corresponding author upon request. AI was not used for any part of this study. Complete disclosure of interest forms according to ICMJE are available on the article page, doi: 10.2340/17453674.2026.46950

## Results

25 patients participated in the study (Figure 2). Patients characteristics showed that the average age was 64 years with a mean BMI just below 29 and preoperative varus alignment in 20 cases (Table 2).

**Figure 2:**
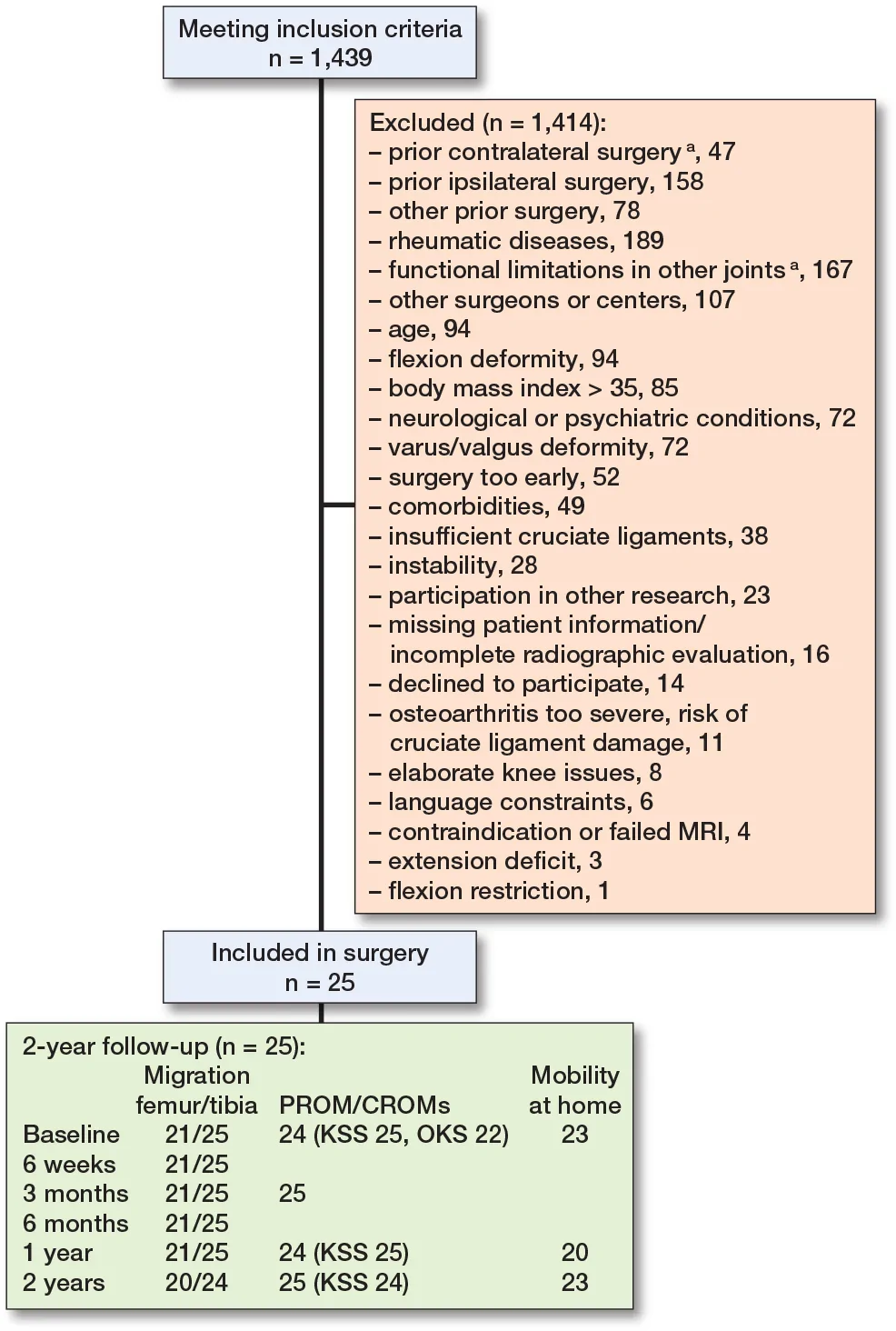
Patient flow diagram illustrating enrollment, exclusions, and follow-up completion. Reasons for exclusion marked with a are criteria only enforced before the amendment.

**Table 2:** Baseline patient characteristics. Values are mean (standard deviation [SD]) or count

Item	Baseline (n = 25)
Age	64 (SD 7.8)
Sex (female / male)	13 / 12
Body mass index	29 (SD 4.4)
Side (left / right)	12 / 13
Preoperative leg alignment	
LDFA (°)	88 (SD 2.1)
MPTA (°)	88 (SD 2.4)
Hip knee ankle angle (°)	
Varus (n = 20)	4 (SD 2.6)
Valgus (n = 5)	2 (SD 1.2)
K&L grade (1 / 2 / 3 / 4)	2 / 1 / 10 / 12

LDFA: lateral distal femoral angle; MPTA: medial proximal tibial angle; K&L: Kellgren & Lawrence

### Primary outcome – RSA

4 femoral components were excluded from analysis: 2 due to a high CN and 2 because too few matching markers were visible to match a model. At 2-year follow-up, 1 additional measurement (femur and tibia) was excluded due to asynchronous acquisition of the images. At 2-year follow-up the mean number of markers for femur and tibia were 5.9 (SD 1.7) and 7.5 (SD 1.5), CNs were 59 (SD 24) and 43 (SD 13), and MEs were 0.18 (SD 0.10) and 0.18 (SD 0.09), respectively.

All migration values are presented as least-squares means, and a full overview of all RSA results can be found in Figures 3 and 4 and Supplementary Table S2 (see Supplementary data).

**Figure 3:**
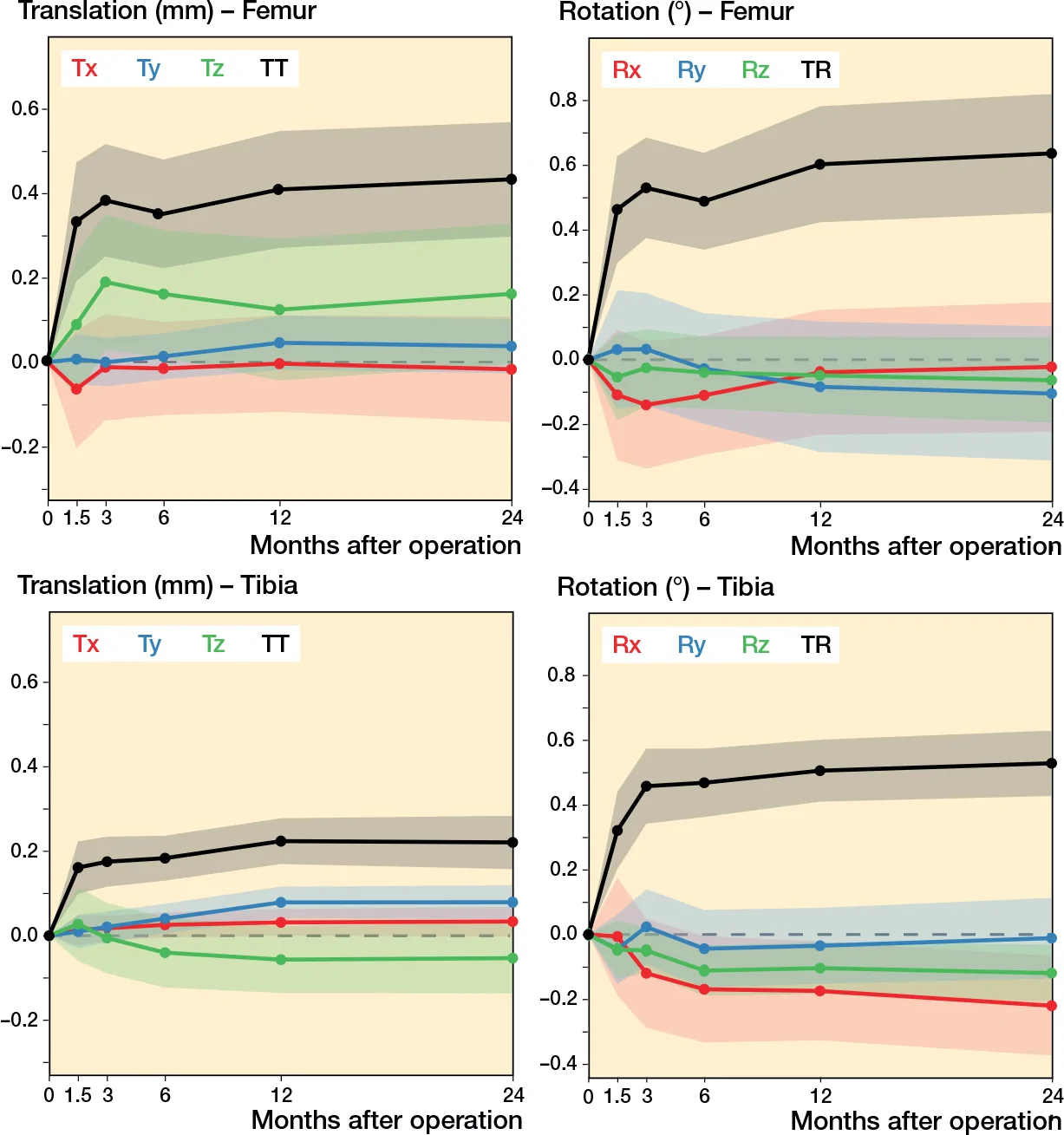
Least-squares means (LS means) with 95% confidence intervals for femoral and tibial translations (Tx, Ty, Tz, total translation [TT]), and rotations (Rx, Ry, Rz, total rotation [TR]) over time, derived from linear mixed-effects models. Measurements are shown at 6 weeks, 3 months, 6 months, 1 year, and 2 years postoperatively; shaded areas represent 95% confidence intervals.

**Figure 4:**
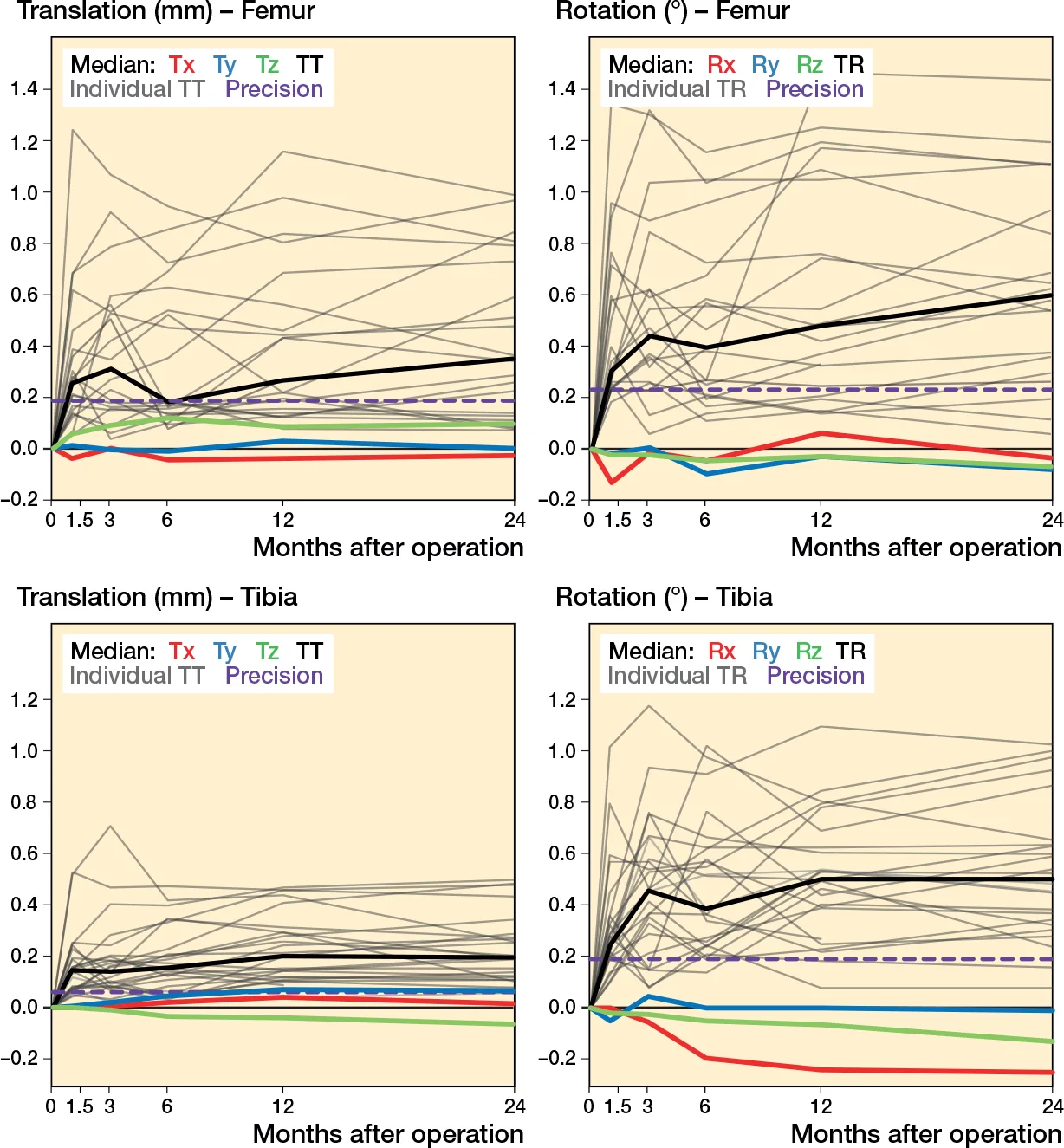
Median translations in mm and rotations in ° of the femoral and tibial components along and around the x, y, and z axis, as well as the total translations (TT), total rotations (TR), and median TT and TR precision (purple dotted line) until 2 years postoperatively. The grey lines present the individual participant’s TT or TR.

#### Tibia

At 2-year follow-up TT was 0.22 mm (CI 0.16–0.28) and TR was 0.53° (CI 0.43–0.63). MTPM_6-months_ was 0.35 mm (CI 0.27–0.43) mm and MTPM_1-year_ was 0.40 mm (CI 0.33–0.48). The model-based ΔMTPM_6–12 months_ was 0.05 mm (CI –0.01 to 0.12) and ΔMTPM_1–2 years_ was 0.02 mm (–0.04; to 0.07). The MTPM-Emax was 0.48 mm and k-value 0.81 months.

#### Femur

At 2-year follow-up TT was 0.43 mm (CI 0.30;–0.57) and TR was 0.64° (CI 0.46–0.82). MTPM_6-months_ was 0.51 mm (CI 0.32–0.70) and MTPM_1-year_ was 0.59 mm (CI 0.38–0.80). The model-based ΔMTPM_6–12 months_ was 0.08 mm (CI −0.04 to 0.19) and ΔMTPM_1–2 years_ was 0.05 mm (CI −0.06–0.16). The MTPM-Emax was 0.81 mm and k-value 0.34 months.

### Secondary outcomes

Linear mixed-effects models showed significant effects of time on gait speed and stride length, not on maximum gait speed or stride time. Compared with preoperative values, gait speed increased at 1 year (Δ = 0.09 m/s, P = 0.008) and 2 years (Δ = 0.07 m/s, P = 0.04), improvements below the MCID of 0.10 m/s. Stride length increased at 1 year (Δ = 0.06 m, P = 0.02), but not at 2 years (Δ = 0.05 m, P = 0.07). No differences were observed between 1-year and 2-year follow-up (Table 3).

**Table 3:** Patient- and clinician-reported outcomes and gait outcomes at all follow-up timepoints, presented as least-squares means and 95% confidence interval

Item	Baseline	3 months	1 year	2 years
Gait outcomes, n	23		20	23
Gait speed (m/s)	1.16 (1.09–1.24)	N/A	1.26 (1.18–1.33)	1.24 (1.16–1.31)
Max. gait speed (m/s)	1.37 (1.31–1.44)	N/A	1.42 (1.35–1.48)	1.42 (1.36–1.48)
Stride time (s)	1.16 (1.12–1.20)	N/A	1.13 (1.09–1.18)	1.13 (1.08–1.17)
Stride length (m)	1.30 (1.23–1.37)	N/A	1.36 (1.29–1.43)	1.35 (1.28–1.42)
Knee Society Score, n	25	25	25	24
Clinical	68 (64–72)	86 (82–89)	94 (90–98)	93 (89–97)
Functional	65 (59–71)	66 (60–72)	83 (77–89)	88 (82–94)
KOOS-PS, n	24	25	24	25
Score	53 (48–58)	56 (52–61)	67 (62–72)	72 (67–76)
Oxford Knee Score, n	22	25	24	25
Score	27 (25–29)	33 (31–35)	41 (39–44)	43 (40–45)
EQ-5D-5L, n	24	25	24	25
Time trade-off	0.6 (0.6–0.7)	0.8 (0.7–0.8)	0.9 (0.8–0.9)	0.9 (0.8–0.9)
Visual analogue scale	71 (66–76)	76 (71–81)	81 (76–86)	85 (80–90)
NPRS, n	24	25	24	25
Rest	4.8 (4.1– 5.6)	3.2 (2.5–4.0)	1.2 (0.5–2.0)	0.8 (0.0–1.5)
Activity	6.8 (6.0–7.6)	4.3 (3.5–5.1)	2.0 (1.2–2.9)	1.6 (0.8–2.5)
Forgotten Joint Score, n		25	24	25
Score	N/A	16 (7–24)	47 (38–55)	58 (50–67)

KOOS-PS: Knee Injury and Osteoarthritis Outcome Score Physical Function; EQ-5D-5L: Euro-Qol 5-dimensions 5-levels; NPRS: Numeric Pain Rating Scale.

Healthy controls had a mean gait speed of 1.31 m/s (CI 1.24–1.38) and maximum gait speed of 1.52 m/s (CI 1.45–1.58) (Figure 5). Preoperatively, the BCR group exhibited lower gait speed (Δ = −0.14 m/s, P = 0.01, above the MCID) and maximum gait speed (Δ = −0.14 m/s, P < 0.01). No differences were observed between the BCR group and healthy controls at 1-year or 2-year follow-up for either gait speed or maximum gait speed, indicating that gait speed was comparable with healthy control levels at these time points. Least-squares means of the median stride time and stride length for the healthy controls were 1.11 s (CI 1.07–1.15) and 1.40 m (CI 1.33–1.46), respectively. There were no differences between the groups in stride time and stride length at any time point.

**Figure 5:**
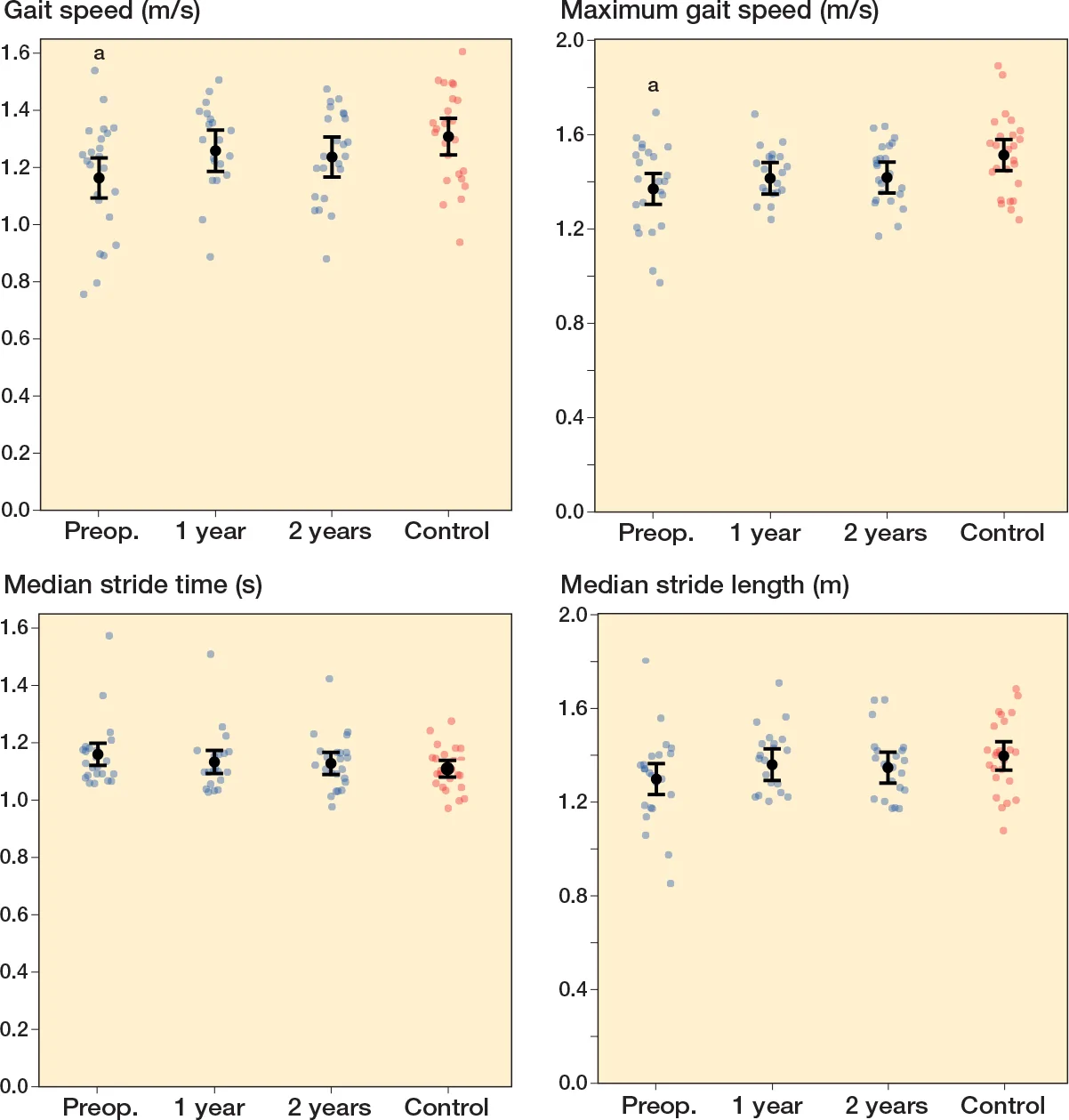
Gait outcome measures over time for the bicruciate-retaining replacement (BCR) and control groups. Individual measurements are shown as jittered points, with group-level least-squares means (BCR) and means (control) with 95% confidence intervals overlaid and an age- and sex-matched control cohort [10].

The BCR group had a significantly higher BMI than healthy controls (28.9 vs 24.5; mean difference 4.4, CI 2.2–6.6, P < 0.001).

All PROMs and clinician-reported outcomes measures (CROMs) improved after surgery (see Table 3). The KSS clinical score increased at 3 months (exceeding the MCID), while KSS functional improvement exceeded the MCID at 1 year. KOOS-PS scores demonstrated progressive improvements in physical function across follow-up, exceeding the MCID between 3 months and 1 year. OKS scores improved above the MCID at 1 year. Health-related quality of life (EQ-5D-5L) improved; pain decreases were above the MCID. FJS indicated increased joint awareness early after surgery, followed by a decline at 1-year and 2-year follow-up above MCID.

### Adverse events

In 2 patients an intraoperative anterior tibial eminence fracture occurred; both were treated with screw fixation and did not require further intervention. 4 patients reported persistent postoperative pain, of whom 1 was referred to a pain management clinic and 1 received additional pain medication. In the other 2 pain-report cases, no clear relation to the study procedure or the implant was observed. 2 patients developed immediate postoperative oedema requiring the use of an elastic compression stocking. In 1 of these patients, the oedema resulted in a limitation of knee flexion (60°) and the same patient also experienced an extension deficit (5–10°), for which physiotherapy and an extension splint were prescribed. This patient reached 110° flexion with an extension deficit of 5° at 2-year follow-up.

## Discussion

This is the first RSA study on the specific implant in our study. The primary aim of our study was to assess the fixation of BCR TKA up to 2 years postoperatively. Secondary aims included the evaluation of clinical and functional outcomes. We found relatively low amounts of TT and TR for both components at 2-year follow-up. Tibial MTPM remained below established RSA TKA benchmark values, with MTPM at 6 months and 1 year below 0.5 mm [24,25] and MTPM-Emax below 1.3 mm [27]. Mean increases in MTPM from 6 months to 1 year and from 1 year to 2 years indicate stabilization in migration for both components [24]. Because established RSA benchmarks are based on MTPM, this measure was used to contextualize the observed migration. Taken together, the findings for TT, TR, and MTPM indicate a pattern of limited migration followed by stabilization over time. Gait speed and stride length improved significantly after surgery, indicating improved function, but remained below the MCID of 0.1 m/s. In addition, patients demonstrated clinically relevant improvements in clinical and functional patient-reported outcomes. AEs included 2 intraoperative tibial eminence fractures, 4 cases of persistent postoperative pain, and 2 cases of postoperative oedema with temporary knee stiffness.

Evidence on this specific implant remains limited. A prospective multicenter cohort study by Yamagami et al. reported good 2-year survivorship and clinically meaningful improvements in patient-reported outcomes, accompanied by stable radiographic findings [28]. However, the authors emphasized the need for further research. To date, RSA studies on this implant are lacking. Comparison with available RSA studies on a different BCR TKA design shows comparable femoral RSA migration [1,3]. Interestingly, the median tibial migration in our study appears slightly lower at 2-year follow-up, with a narrower IQR [3,29]. Furthermore, Mills et al. [1] reported a diverging rotational migration pattern up to 2-year follow-up, which was not observed in our study. This may suggest earlier stabilization of the tibial component and less within-group variability in the current cohort.

These differences may be explained by 2 main factors. First, the implant design: the implant used in this study features a wide, relatively deep keel terminating in 2 medium-sized pegs, along with 4 additional smaller pegs. In contrast, the implant used in the other studies [1,29] incorporated only 4 pegs, comparable in size to the 2 medium-sized pegs in our study, to provide fixational support. Second, the surgical technique: implanting a BCR TKA is technically challenging, as preservation of both cruciate ligaments reduces the available surgical space and requires precise soft-tissue balancing. In our study, the BCR TKA was implanted using PSIs, which aim to standardize and optimize implant alignment and positioning. This may have contributed to the smaller within-group variability observed in this study and may potentially improve early stabilization of migration.

Based on the double examinations, the femoral component showed lower measurement precision compared with the tibial component. This may be related to the more rounded geometry of the femoral component, as well as greater local variability in its surface contour and finishing, which may make model-based RSA matching more challenging. In contrast, the sharper and more distinct geometric boundaries of the tibial component may provide more robust features for model matching. Nevertheless, similar femoral precision has been reported by Mills et al. [1], suggesting that this may be an inherent characteristic of model-based RSA assessment of femoral TKA components rather than a study-specific finding, thereby supporting the comparison of femoral migration measurements between studies.

Our study demonstrated increases in gait speed after surgery (below MCID), as well as improvements in PROMs exceeding predefined MCIDs. Interestingly, a study conducted during the same period at our institution investigating cruciate-retaining (CR) implants reported a similar recovery in daily-life walking performance, as well as comparable scores on KOOS-PS and NRS pain [30]. These comparable findings suggest that the observed improvements may not be specific to the BCR design, although direct comparison between matched cohorts would be needed to determine whether BCR provides an additional functional benefit over conventional implant designs.

A recent literature review indicated that clear indications or guidelines regarding the use of BCR TKA remain lacking [4]. However, it appears evident that the indication for this procedure is relatively narrow, which was also reflected in this study by the high number of exclusions. BCR TKA requires intact cruciate ligaments, and most surgeons also require limited varus/valgus deformity and flexion contracture. In addition, many surgeons apply restrictions regarding patient age (> 80 years) and BMI (> 34.9) [31]. As a result, only a small subset of TKA patients qualify for this procedure. Part of this population, patients with isolated compartmental OA, is also the target population for unicompartmental knee arthroplasty. This further reduces the already limited eligible population. This restricted indication range may partly explain why clinical experience and high-quality evidence remain scarce for BCR TKA designs.

In our study, BCR TKA was implanted using mechanical alignment, which was the established alignment approach when the study was initiated. Although patient-specific alignment philosophies have gained increasing interest in recent years, their potential benefits in BCR TKA remain uncertain, particularly in the highly selected patient population included in our study. Further research is needed to determine whether alternative alignment philosophies provide additional clinical benefit while maintaining adequate implant fixation and survivorship.

### Limitations

First, no direct comparison with a control group was possible. Therefore, results were compared with existing RSA benchmarks. These benchmarks are based on CR or PS TKA designs, apply only to tibial components, and may vary depending on implant design and fixation method. Thereby, they are based on MTPM and cannot be directly used as a reference value for TT and TR. Second, patient inclusion was slower than anticipated, resulting in an inclusion period of 3 years. To facilitate recruitment, the exclusion criteria for the gait assessment were amended during the inclusion period, which may have introduced additional heterogeneity within the study population. Third, gait outcomes were compared with healthy controls who had originally been included in a separate study using the same measurement protocol, resulting in suboptimal matching. Lastly, this was a single-center study with a relatively small sample size. In addition, all procedures were performed by experienced surgeons, which may limit generalizability of the findings to less experienced centers.

### Conclusion

RSA analysis of this BCR TKA demonstrated relatively low TT and TR for both components. Tibial MTPM was below established TKA benchmark values. This indicates good early fixation at 2-year follow-up. These findings were accompanied by clinically meaningful improvements in PROMs. There were also improvements in gait speed, which appear to be of limited clinical relevance.

### Supplementary data

Supplementary Tables S1–S2 are available as supplementary data on the article page, doi: 10.2340/17453674.2026.46950

## Supplementary Material



## Notes

KM: formal analysis, investigation, data curation, writing—original draft, visualization, and project administration. SS: investigation, writing—review & editing, and project administration. KD, SvL: investigation and writing—review & editing. PH: conceptualization, methodology, writing—review & editing, supervision, and funding acquisition.

The authors would like to thank Jolanda Rubrech-van As for data management and Katrijn Smulders, Ramon Boekesteijn, and Frank Bruning for their contribution to the at-home gait monitoring.

Handling co-editors: Paul Gerdhem and Robin Christensen

*Acta* thanks Stephan Maximilian Röhrl and Frank-David Øhrn for help with peer review of this manuscript.
