# Supplementary material for: Stable 2-year migration on radiostereometric analysis and improved gait speed and function after bicruciate-retaining TKA: a prospective cohort of 25 patients
Source: Acta Orthop. 2026 Oct 26;97:767–75. doi: 10.2340/17453674.2026.46950 (PMC13697300; doi:10.2340/17453674.2026.46950)
Supplement: Supplementary file 1 [file ActaO-97-46950-s1.pdf]

Supplementary data

Table S1. Full overview of all inclusion and exclusion criteria

Inclusion	Exclusion
Non-inflammatory knee OA, confirmed by radiology	BMI > 35
Set to receive primary cemented TKA	Major non-arthroscopic surgery to the study knee, included HTO
Age between 40 and 80 years, inclusive, on the day of surgery	Previous hip replacement surgery, or other joint replacements influencing gait or is planning on this in the next 6–12 months
Plans to be available for follow-up until 2 years postoperatively	Moderate to severe pain in contralateral knee, hip or ankle, defined as an average score of > 4 on items 3–6 of the Short Brief Pain Inventory
Stable health (ASA score ≤ 3) and is free of or treated for cardiac, pulmonary, hematological, or other conditions that would pose excessive operative risk	Active, local infection or systemic infection
Correctable or < 5 degrees rigid (non-correctable) varus or valgus deformity of the knee	Prior high-energy trauma to the affected knee or prior history of anterior and/or posterior cruciate ligament rupture
OA is unilateral or bilateral with the contralateral knee functioning properly, not operated on in the last 6 months and not planned for TKA in the coming 24 months	Suspicion of anterior and/or posterior cruciate ligament rupture at clinical examination or on radiographs
Anteromedial or posterolateral end-stage OA on lateral radiograph, without posteromedial osteo-phytes (indicating insufficient cruciate ligaments)	Documented osteoporosis with patient in active medical treatment
Participants must be able to give informed consent	Physical, emotional, or neurological conditions that would compromise compliance with postoperative rehabilitation and follow-up
	Bone quality compromised by disease or infection which cannot provide adequate support and/or fixation to the prosthesis
	Severe instability of the knee joint secondary to the absence of collateral ligament integrity and function
	Knee flexion < 90°
	Fixed flexion deformity > 5° (passive extension lag)
	> 10 degrees extension deficit (active restraint to extension)
	No proper functioning patellar tendon on the affected side; measured as inability of active extension of the knee
	Impaired extensor strength defined as MRC grade < 4
	Any musculoskeletal or neurological disease, or uncorrected visual impairment other than osteoarthritis that impairs gait or balance
	Incomplete or insufficient tissue surrounding the study knee
	Severe damage to any knee ligament other that the ACL
	Undergone a patellectomy in the study knee
	Osteophytes in the intercondylar notch on AP radiograph
	Contra-indication for MRI
	Substantial lateral tibial subluxation
	Rheumatoid arthritis, any auto-immune disorder, immunosuppressive disorder, or a terminal illness

Table S2. Migration outcomes, including translations and rotations along and around the x, y, and z axis, total translation (TT) and total rotation (TR), and maximum total point motion (MTPM), for tibial and femoral components at all follow-up timepoints referenced to the baseline measurement. Medians and interquartile ranges are presented as well as least-squares (LS) means and corresponding 95% confidence intervals (CI) estimated from mixed-models for repeated measures. For MTPM the data was first log-transformed before fitting the model and subsequently back-transformed

Item		6 weeks	3 months	6 months	1 year	2 years
Tibia		n = 25	n = 25	n = 25	n = 25	n = 24
TT (mm)	LS mean (CI)	0.16 (0.10 to 0.22)	0.18 (0.12 to 0.24)	0.18 (0.13 to 0.24)	0.22 (0.17 to 0.28)	0.22 (0.16 to 0.28)
	Median (IQR)	0.14 (0.07 to 0.19)	0.14 (0.08 to 0.20)	0.15 (0.08 to 0.23)	0.20 (0.12 to 0.29)	0.19 (0.11 to 0.27)
Tx (mm)	LS mean (CI)	0.01 (–0.02 to 0.05)	0.02 (–0.01 to 0.05)	0.03 (–0.00 to 0.06)	0.03 (–0.00 to 0.06)	0.03 (–0.00 to 0.07)
	Median (IQR)	–0.00 (–0.03 to 0.06)	0.00 (–0.02 to 0.08)	0.02 (–0.02 to 0.07)	0.04 (–0.03 to 0.09)	0.01 (–0.03 to 0.08)
Ty (mm)	LS mean (CI)	0.01 (–0.03 to 0.05)	0.02 (–0.02 to 0.06)	0.04 (0.01 to 0.08)	0.08 (0.04 to 0.12)	0.08 (0.04 to 0.12)
	Median (IQR)	0.00 (–0.02 to 0.03)	0.02 (–0.01 to 0.06)	0.04 (–0.00 to 0.08)	0.07 (0.03 to 0.13)	0.06 (0.01 to 0.12)
Tz (mm)	LS mean (CI)	0.03 (–0.06 to 0.11)	–0.01 (–0.09 to 0.08)	–0.04 (–0.12 to 0.04)	–0.06 (–0.14 to 0.02)	–0.05 (–0.14 to 0.03)
	Median (IQR)	–0.00 (–0.07 to 0.08)	–0.01 (–0.15 to 0.06)	–0.04 (–0.18 to 0.05)	–0.04 (–0.21 to 0.05)	–0.07 (–0.17 to 0.08)
TR (°)	LS mean (CI)	0.32 (0.20 to 0.44)	0.46 (0.34 to 0.57)	0.47 (0.36 to 0.57)	0.51 (0.41 to 0.60)	0.53 (0.43 to 0.63)
	Median (IQR)	0.24 (0.20 to 0.36)	0.45 (0.29 to 0.58)	0.38 (0.24 to 0.62)	0.50 (0.39 to 0.62)	0.50 (0.34 to 0.64)
Rx (°)	LS mean (CI)	–0.01 (–0.19 to 0.18)	–0.12 (–0.29 to 0.05)	–0.17 (–0.33 to –0.01)	–0.17 (–0.33 to –0.02)	–0.22 (–0.37 to –0.07)
	Median (IQR)	–0.00 (–0.18 to 0.10)	–0.06 (–0.37 to 0.11)	–0.20 (–0.44 to 0.07)	–0.24 (–0.41 to 0.10)	–0.25 (–0.47 to 0.03)
Ry (°)	LS mean (CI)	–0.05 (–0.15 to 0.06)	0.02 (–0.09 to 0.14)	–0.04 (–0.16 to 0.08)	–0.03 (–0.15 to 0.08)	–0.01 (–0.14 to 0.11)
	Median (IQR)	–0.05 (–0.12 to 0.18)	0.04 (–0.11 to 0.26)	–0.00 (–0.24 to 0.13)	–0.00 (–0.15 to 0.13)	–0.01 (–0.22 to 0.16)
Rz (°)	LS mean (CI)	–0.05 (–0.14 to 0.04)	–0.05 (–0.12 to 0.02)	–0.11 (–0.19 to –0.03)	–0.10 (–0.18 to –0.02)	–0.12 (–0.21 to –0.03)
	Median (IQR)	–0.02 (–0.10 to 0.03)	–0.03 (–0.16 to 0.06)	–0.05 (–0.24 to 0.04)	–0.07 (–0.24 to 0.04)	–0.13 (–0.25 to 0.03)
MTPM (mm)	LS mean (CI)	0.27 (0.20 to 0.34)	0.33 (0.26 to 0.41)	0.35 (0.27 to 0.43)	0.40 (0.33 to 0.48)	0.42 (0.33 to 0.51)
	Median (IQR)	0.28 (0.20 to 0.35)	0.37 (0.25 to 0.46)	0.32 (0.26 to 0.53)	0.40 (0.31 to 0.59)	0.44 (0.32 to 0.64)
Femur		n = 21	n = 21	n = 21	n = 21	n = 20

TT (mm)	LS mean (CI)	0.33 (0.19 to 0.47)	0.38 (0.25 to 0.52)	0.35 (0.22 to 0.48)	0.41 (0.27 to 0.55)	0.43 (0.30 to 0.57)
	Median (IQR)	0.25 (0.15 to 0.38)	0.31 (0.16 to 0.53)	0.18 (0.14 to 0.54)	0.27 (0.15 to 0.56)	0.35 (0.13 to 0.75)
Tx (mm)	LS mean (CI)	–0.06 (–0.20 to 0.08)	–0.01 (–0.14 to 0.11)	–0.02 (–0.13 to 0.09)	–0.00 (–0.12 to 0.11)	–0.02 (–0.14 to 0.11)
	Median (IQR)	–0.04 (–0.12 to 0.05)	0.00 (–0.03 to 0.14)	–0.05 (–0.08 to 0.10)	–0.04 (–0.08 to 0.08)	–0.03 (–0.13 to 0.05)
Ty (mm)	LS mean (CI)	0.01 (–0.05 to 0.07)	–0.00 (–0.06 to 0.06)	0.01 (–0.04 to 0.07)	0.05 (–0.02 to 0.11)	0.04 (–0.03 to 0.10)
	Median (IQR)	0.01 (–0.04 to 0.06)	–0.01 (–0.04 to 0.05)	–0.02 (–0.06 to 0.04)	0.03 (–0.03 to 0.11)	0.00 (–0.06 to 0.09)
Tz (mm)	LS mean (CI)	0.09 (–0.08 to 0.26)	0.19 (0.03 to 0.35)	0.16 (0.01 to 0.31)	0.12 (–0.04 to 0.29)	0.16 (–0.01 to 0.33)
	Median (IQR)	0.05 (–0.12 to 0.21)	0.09 (–0.03 to 0.42)	0.11 (–0.05 to 0.44)	0.08 (–0.13 to 0.41)	0.09 (–0.07 to 0.52)
TR (°)	LS mean (CI)	0.47 (0.30 to 0.63)	0.53 (0.38 to 0.69)	0.49 (0.34 to 0.64)	0.61 (0.43 to 0.79)	0.64 (0.46 to 0.82)
	Median (IQR)	0.30 (0.24 to 0.59)	0.44 (0.26 to 0.62)	0.39 (0.21 to 0.67)	0.48 (0.24 to 1.04)	0.60 (0.34 to 0.97)
Rx (°)	LS mean (CI)	–0.11 (–0.31 to 0.09)	–0.14 (–0.34 to 0.06)	–0.11 (–0.29 to 0.07)	–0.04 (–0.23 to 0.15)	–0.02 (–0.22 to 0.18)
	Median (IQR)	–0.14 (–0.23 to 0.12)	–0.01 (–0.44 to 0.07)	–0.05 (–0.31 to 0.03)	0.06 (–0.35 to 0.24)	–0.03 (–0.39 to 0.34)
Ry (°)	LS mean (CI)	0.03 (–0.15 to 0.22)	0.03 (–0.14 to 0.21)	–0.03 (–0.20 to 0.14)	–0.08 (–0.29 to 0.12)	–0.10 (–0.31 to 0.10)
	Median (IQR)	–0.02 (–0.20 to 0.20)	0.00 (–0.18 to 0.19)	–0.10 (–0.23 to 0.04)	–0.03 (–0.38 to 0.06)	–0.08 (–0.42 to 0.09)
Rz (°)	LS mean (CI)	–0.05 (–0.19 to 0.08)	–0.03 (–0.15 to 0.09)	–0.04 (–0.15 to 0.07)	–0.05 (–0.17 to 0.07)	–0.06 (–0.20 to 0.07)
	Median (IQR)	–0.03 (–0.15 to 0.04)	–0.02 (–0.08 to 0.12)	–0.05 (–0.15 to 0.06)	–0.03 (–0.19 to 0.04)	–0.07 (–0.24 to 0.11)
MTPM (mm)	LS mean (CI)	0.51 (0.30 to 0.72)	0.55 (0.35 to 0.76)	0.51 (0.32 to 0.70)	0.59 (0.38 to 0.80)	0.64 (0.41 to 0.86)
	Median (IQR)	0.44 (0.33 to 0.80)	0.48 (0.34 to 1.05)	0.43 (0.29 to 1.01)	0.52 (0.34 to 1.04)	0.68 (0.34 to 1.22)
